# Cost-effectiveness of a Smoking Cessation Intervention for Parents in Pediatric Primary Care

**DOI:** 10.1001/jamanetworkopen.2021.3927

**Published:** 2021-04-01

**Authors:** Olivier Drouin, Ryoko Sato, Jeremy E. Drehmer, Emara Nabi-Burza, Bethany Hipple Walters, Jonathan P. Winickoff, Douglas E. Levy

**Affiliations:** 1 Division of General Pediatrics, Department of Pediatrics, CHU Sainte-Justine, Montréal, Quebec, Canada; 2 Department of Social and Preventive Medicine, Université de Montréal School of Public Health, Montréal, Québec, Canada; 3 Harvard T.H. Chan School of Public Health, Boston, Massachusetts; 4 Division of General Academic Pediatrics, Massachusetts General Hospital for Children, Boston; 5 Tobacco Research and Treatment Center, Massachusetts General Hospital, Boston; 6 Harvard Medical School, Boston, Massachusetts; 7 Julius B. Richmond Center of Excellence, American Academy of Pediatrics, Itasca, Illinois; 8 Mongan Institute Health Policy Research Center, Massachusetts General Hospital, Boston

## Abstract

**Importance:**

Parental smoking adversely affects parents’ and children’s health. There are effective interventions delivered in pediatric settings to help parents quit smoking. The cost-effectiveness of this type of intervention is not known.

**Objective:**

To evaluate the cost-effectiveness of a parental smoking cessation intervention, the Clinical Effort Against Secondhand Smoke Exposure (CEASE) program, delivered in pediatric primary care, compared with usual care from a health care organization’s perspective.

**Design, Setting, and Participants:**

This economic evaluation used data on intervention costs and parental smoking cessation collected prospectively as part of the CEASE randomized clinical trial. Data were collected at pediatric offices in 5 US states from April 2015 to October 2017. Participants included parents of children attending 10 pediatric primary care practices (5 control, 5 intervention). Data analysis was performed from October 2019 to August 2020.

**Exposures:**

The trial compared CEASE (practice training and support to address family tobacco use) vs usual care.

**Main Outcomes and Measures:**

The overall cost and incremental cost per quit of the CEASE intervention were calculated using microcosting methods. CEASE effectiveness was estimated using 2 trial outcomes measures assessed in repeated cross-sections: (1) change in smoking prevalence assessed by parental report for intervention vs usual care practices at 2 weeks after program initiation (baseline) and at 2-year follow-up and (2) changes in the proportion of smokers who achieved cotinine-confirmed smoking cessation in the previous 2 years at baseline vs follow-up. Monte Carlo analyses were used to provide 95% CIs.

**Results:**

The study included a total of 3054 participants (1523 at baseline and 1531 at follow-up); 2163 (70.8%) were aged 25 to 44 years old, and 2481 (81.2%) were women. Over 2 years, the total cost of implementing and sustaining CEASE across 5 intervention practices was $115 778. The incremental cost per quit for CEASE compared with usual care was $1132 (95% CI, $653-$3603), according to the change in parent-reported smoking prevalence, and $762 (95% CI, $418-$2883), according to cotinine-confirmed cessation. CEASE was cost-effective at a willingness-to-pay threshold of $2000 per quit in 88.0% of simulations based on the parent-reported smoking prevalence and 94.6% of simulations based on cotinine-confirmed smoking cessation measures.

**Conclusions and Relevance:**

These findings suggest that the CEASE intervention was associated with an incremental cost per quit that compared favorably with those of other clinical smoking cessation interventions. CEASE is inexpensive to initiate and maintain in the clinical pediatric setting, suggesting that it has the potential for a high impact on population health.

## Key Points

**Question:**

Is the Clinical Effort Against Secondhand Smoke Exposure (CEASE) intervention a cost-effective way for pediatric practices to help parents quit smoking?

**Findings:**

In this economic evaluation, the CEASE intervention integrated into pediatric primary care practices had an incremental cost-effectiveness ratio of $1132 per quit. CEASE was cost-effective at a willingness-to-pay threshold of $2000 per quit in 88.0% of simulations based on changes in parent-reported smoking prevalence.

**Meaning:**

These findings suggest that the cost-per-quit of CEASE compares favorably to those of other smoking cessation interventions in the clinical setting.

## Teaser

This economic evaluation examines the cost-effectiveness of a parental smoking cessation intervention delivered in pediatric primary care, compared with usual care, from a health care organization’s perspective.

## Introduction

Tobacco smoke exposure remains a major cause of morbidity in the pediatric population, and parental smoking cessation has major benefits for both child and adult health.^1^ Screening families for tobacco use, providing counseling, and referring family members who smoke to services to assist them with smoking cessation is recommended by national guidelines in the US,^2^ but adherence to those guidelines is low.^3^ Direct costs of parental tobacco smoking on children’s health were previously estimated at $1.9 billion per year in the US.^4^ The overall economic cost of smoking in the US is estimated at more than $300 billion annually.^5^ Parents who quit will save an average of more than 10 years of life, and quitting before the age of 40 years can reduce smoking-related morbidity and mortality by 90%.^6^

System-level interventions are needed to improve screening rates and provision of meaningful cessation assistance if significant population health improvements are to be expected.^2^ However, stakeholders such as policy makers and other health care decision-makers need data about costs and health benefits to make evidence-informed decisions about investments in such programs. The Clinical Effort Against Secondhand Smoke Exposure (CEASE) intervention provides a framework for systematically screening families in pediatric primary care practices for tobacco use and delivering evidence-based cessation assistance.^7^ In prior research,^8^ CEASE was associated with decreased smoking prevalence and increased smoking cessation among parents whose children had received pediatric care. One response to these promising findings was concern about the resource and economic burden CEASE might impose on pediatric practices.^9^ In this economic evaluation, we describe a prespecified analysis quantifying the total costs and incremental cost-effectiveness of CEASE from the perspective of a health care organization.

## Methods

This report follows the Consolidated Health Economic Evaluation Reporting Standards (CHEERS) guideline for economic evaluations.^10^ This study was a secondary data analysis of the CEASE trial, which was approved by the institutional review boards of the American Academy of Pediatrics, the Massachusetts General Hospital, and individual practices, when required. Participants in the CEASE trial provided written informed consent, so we did not seek it again.

### CEASE Cluster Randomized Clinical Trial

CEASE was evaluated in a cluster randomized clinical trial conducted among 10 pediatric primary care practices located in 5 states (Indiana, North Carolina, Ohio, Tennessee, and Virginia, with 1 intervention practice and 1 control practice per state) between April 2015 and October 2017. Participating practices were recruited by the American Academy of Pediatrics. Practices were considered for inclusion if they had a minimum parental smoking prevalence of 15% and served at least 50 families per day. Further study details are reported elsewhere.^8^

CEASE focused on training and providing program management support to clinicians and clinic staff to address family tobacco use.^8^ In intervention practices, families were screened for tobacco use at check-in using a tablet computer handed to them by clinic staff.^8^ When a household member was identified as a smoker, office staff gave them the CEASE Action Sheet, which included prescriptions for nicotine replacement therapy (NRT), offered to connect them with the state’s smoking cessation telephone hotline (referred to hereafter as a *quitline*), provided instructions to use a free text messaging–based cessation support service,^11^ and presented evidence-based recommendations to clinicians for providing smoking cessation counseling.^12^ Practices were asked to fax quitlines referral forms daily; quitlines provided monthly reports to the practices and the study team about referrals received, which were discussed as needed. The study team reviewed reports monthly and offered implementation support. Participants in control practices received usual care.

Estimates of the CEASE intervention’s effectiveness in the current economic evaluation were taken from the CEASE trial.^8^ There were 2 smoking outcomes reported, each based on cross-sectional assessments obtained at baseline and 2-year follow-up. The first was the change in practice-level parent-reported smoking prevalence, and the second was the change in the prevalence of parent-reported, cotinine-confirmed smoking cessation. For cessation, parents were asked whether they had quit smoking over the prior 2 years. The change in parent-reported smoking prevalence over the 2 years of intervention implementation was greater in the intervention than usual care practices (−2.7% vs 1.1%; difference −3.7%; 95% CI, −6.3% to −1.2%).^8^ Similarly, the prevalence of cotinine-confirmed smoking cessation was greater in the intervention than usual care practices (2.4% vs −3.2%; difference, 5.5%; 95% CI, 1.4% to 9.6%).^8^

### Cost Calculation

All costs are reported in 2018 US dollars. This analysis was conducted from the perspective of a health care organization or accountable care organization, an increasingly common care and payment model in the US, assessing the incremental cost of implementing and sustaining CEASE vs usual care. Estimates for costs and effects of these 2 scenarios were drawn from data collected prospectively during the CEASE trial. We did not have data on changes in health care utilization and expenditures, so instead we focused solely on intervention costs, later putting these into the context of potential reductions in health care expenditures. Our primary analysis measured cumulative costs over 24 months, separating fixed and variable costs. As is the case with any multiinstitution implementation effort, there was variability across sites in how the program was implemented, reflecting physical space, staffing, workflows, and preferences. Local costs were captured in their entirety and reflected the intervention as implemented rather than as designed.

#### Personnel

Personnel costs included employee time (wages plus fringe) necessary for fixed and variable costs. Wages were based on 2018 national averages from the US Bureau of Labor statistics (eTable 1 in the Supplement).^13^ Fixed costs included preintervention implementation activities, including peer-to-peer training with practice leaders (approximately 1 hour), training video viewing, a whole-office training call (with clinical and support staff), and those related to UMass Tobacco Treatment Specialist (TTS) training, which was offered to all clinicians and staff. Postimplementation (variable) costs included monthly report preparation and check-in calls with practices, time spent by clinic staff managing tablet computer distribution to families, and time required to handle and fax referrals to state quitlines. There was 1 additional whole-office check-in call after the first year of implementation. One practice hired a part-time employee to contact families identified during the intervention as having a least 1 family member who smoked. Cost details are reported in eTable 2 in the Supplement.

It is assumed that outside the context of a trial, practices would receive programmatic support from the national CEASE office. Support costs consist of salary and fringe for a national coordinator (1 hour per month per practice) and local coordinators (1 hour per week per practice).

#### Supplies and Infrastructure

At intervention practices, letters were sent to 1100 families to follow up on their desired help for smoking cessation services (NRT prescription and/or quitline referral). With the expectation that local pharmacies might not be familiar with prescribed over-the-counter NRT, the CEASE team conducted outreach. Local pharmacies received informational phone calls and letters during the trial to remind them that over-the-counter NRT is covered by many insurance plans and to provide notice that they may receive additional volume of prescriptions for NRT. These efforts were valued on the basis of the cost of materials, postage, and personnel effort for mailing the letters. Additional costs included the purchase of tablet computers and 2-year cellular data plans that were used for screening of parents.

### Statistical Analysis

#### Cost-effectiveness Analysis

Cost-effectiveness was measured from a health system perspective. The main outcome was the incremental cost-effectiveness ratio (ICER), defined as the difference in per-smoker costs between intervention and usual care practices, divided by the incremental effect (change in parent-reported smoking prevalence for intervention vs usual care practices). As an alternative, we calculated the ICER using cotinine-confirmed smoking cessation as the outcome. Because tobacco treatment activity was minimal under usual care (0.1% of parents reported receiving meaningful tobacco treatment), the cost of tobacco treatment under usual care was conservatively assumed to be $0, and the incremental cost of the intervention was simply the implementation cost in the intervention practices.^8^

#### Sensitivity Analyses

Probabilistic sensitivity analyses were conducted using Monte Carlo simulation methods to aggregate the statistical uncertainty and natural variability in model parameters. For each simulation, parameters were drawn from appropriate probability distributions. In each simulation, costs were aggregated using parameters drawn for that iteration, and we calculated incremental costs, effectiveness, and cost-effectiveness. This process was repeated over 10 000 simulations. Confidence intervals for ICERs were determined using the 2.5th and 97.5th percentiles of the simulation estimates. The statistical distributions used for each parameter are detailed in eTable 3 in the Supplement. Distributions were based on primary trial data, where available. Otherwise, we assumed quantities varied randomly, generally with 99% CIs within 10% greater than or less than the base case value (eTable 3 in the Supplement).

To guide individual stakeholders, the proportion of simulations that were cost-effective according to different willingness-to-pay thresholds were modeled. This provides an indication of how likely the intervention is to be cost-effective depending on a given system’s willingness to pay for the intervention.

In addition to probabilistic sensitivity analyses, 1-way deterministic sensitivity analyses were conducted to examine how different assumptions regarding program implementation would affect study findings. The base-case analysis includes the cost of TTS training for practice staff and the cost of direct parent outreach. In practice, the TTS training was unevenly adopted: 2 sites did not participate in any TTS training, 2 sites trained 2 staff members each, and 1 site had 2 staff members and 4 pediatricians participate in the training. One site hired a master’s level staff member to call parents to follow up about the use of smoking cessation services (NRT and/or quitline referral). This was not part of the official study design. On the basis of preliminary evidence suggesting no variation in treatment efficacy across sites, the cost of these 2 efforts was removed in a sensitivity analysis.

Baseline smoking rates in practices varied from 12.1% to 48.5%, with a combined smoking rate of 23.9% in the usual care practices and 27.1% in the intervention practices.^8^ Given the presence of some fixed costs, the cost per smoker (and therefore the cost per quit) could be higher in states or practices with lower smoking rates. Therefore, additional sensitivity analyses estimated how the ICER would vary given different baseline smoking rates. Given the presence of fixed start-up costs and regular ongoing costs, cost-effectiveness was also assessed for varying durations of implementation. Aggregate costs were calculated using Excel software version 16.46 (Microsoft), and simulations were performed using Stata statistical software version 15.1 (StataCorp). Data analysis was performed from October 2019 to August 2020.

## Results

### Total Costs

The study included a total of 3054 participants (1523 at baseline and 1531 at follow-up); 2163 (70.8%) were aged 25 to 44 years old, and 2481 (81.2%) were women. According to previsit screening data in intervention practices over the 24-month study period, it is estimated that approximately 2764 unique smokers (95% CI, 2501-3032 smokers) were screened. Overall program costs for the 24-month study period were $115 778 across the 5 intervention practices (Table 1). There was no cost in the usual care practices. Fixed costs ($20 466) were smaller than variable costs ($95 312) when calculated over a 24-month period. The largest contributions to program costs in the intervention practices were the time spent by front desk staff to manage the electronic tablets used for screening ($23 063) and time spent by study staff to provide programmatic support to practices ($49 509). Overall, wages represented 88% of the total cost. Costs per unique smoker screened over the 24 months were $42 (95% CI, $36-$52) for intervention practices (incremental cost per smoker screened).

**Table 1.** Total Costs for 5 Intervention Sites Over 24-Month Study Period^a^

Type of cost	Cost, $	
	Observed results, intervention	Probabilistic sensitivity analysis results, median (95% CI)^b^
Fixed costs		
Preimplementation		
Peer-to-peer training	1742	1738 (1458-2042)
Training video viewing time	433	434 (199-669)
Whole office training call	1219	1208 (503-1934)
Tablet computers	7466	NA
UMass TTS Training	8898	8832 (8241-9451)
Postimplementation		
Check-in call (1st mo)	501	501 (453-550)
Informational calls to local pharmacies	238	238 (212-267)
Informational letters to local pharmacies	38	38 (35-40)
Subtotal for fixed costs	20 535	20 459 (19 295-21 673)
Variable costs (for 2 y)		
Monthly report preparation	1920	1919 (1743-2109)
Tablet computers data plan	4596	NA
Check-in calls	36	36 (33-40)
In office tablet management (with parents)	24 709	23 152 (12 407-47 239)
In-office tablet management (end of each day)	1717	1716 (1552-1883)
Parent letters	965	965 (893-1040)
Parent calls (1 practice only)	9336	9302 (7415-11 419)
Quitline report management time	3005	3004 (2721-3304)
1-y Whole-office call	1283	1276 (727-1837)
Programmatic support	49 509	49 439 (44 259-55 005)
Subtotal for variable costs (for 2 y)	97 078	95 686 (82 124-120 619)
Total for all costs	117 613	116 168 (102 081-141 234)

Abbreviations: NA, not applicable; TTS, Tobacco Treatment Specialist.

^a^ All costs are reported in 2018 US dollars.

^b^ The 95% CIs were determined using 2.5th and 97.5th percentiles of the simulation estimates.

### Cost-effectiveness

With a risk difference in parent-reported smoking prevalence of −3.7% (95% CI, −6.3% to −1.2%) between intervention and usual care practices,^8^ the ICER for the CEASE intervention was $1132 (95% CI, $653 to $3603) over the 24-month study period. The ICER calculated using cotinine-confirmed smoking cessation was $762 (95% CI, $418 to $2883) (Table 2). Scatter plots illustrating the joint variability of cost and smoking outcomes across probabilistic sensitivity analysis simulations are reported in the eFigure in the Supplement.

**Table 2.** Cost-effectiveness Over 24-Month Study Period^a^

Effectiveness measure	Incremental cost per smoker screened (95% CI), $	Adjusted risk difference, % (95% CI)	ICER (95% CI), $
Parent-reported smoking prevalence	41.89 (36.05 to 52.17)	−3.7 (−6.3 to −1.2)	1132 (653 to 3603)
Cotinine-confirmed smoking cessation	41.89 (36.05 to 52.17)	5.5 (1.4 to 9.6)	762 (418 to 2883)

Abbreviation: ICER, incremental cost-effectiveness ratio.

^a^ All costs are reported in 2018 US dollars.

### Sensitivity Analyses

**Figure 1.**
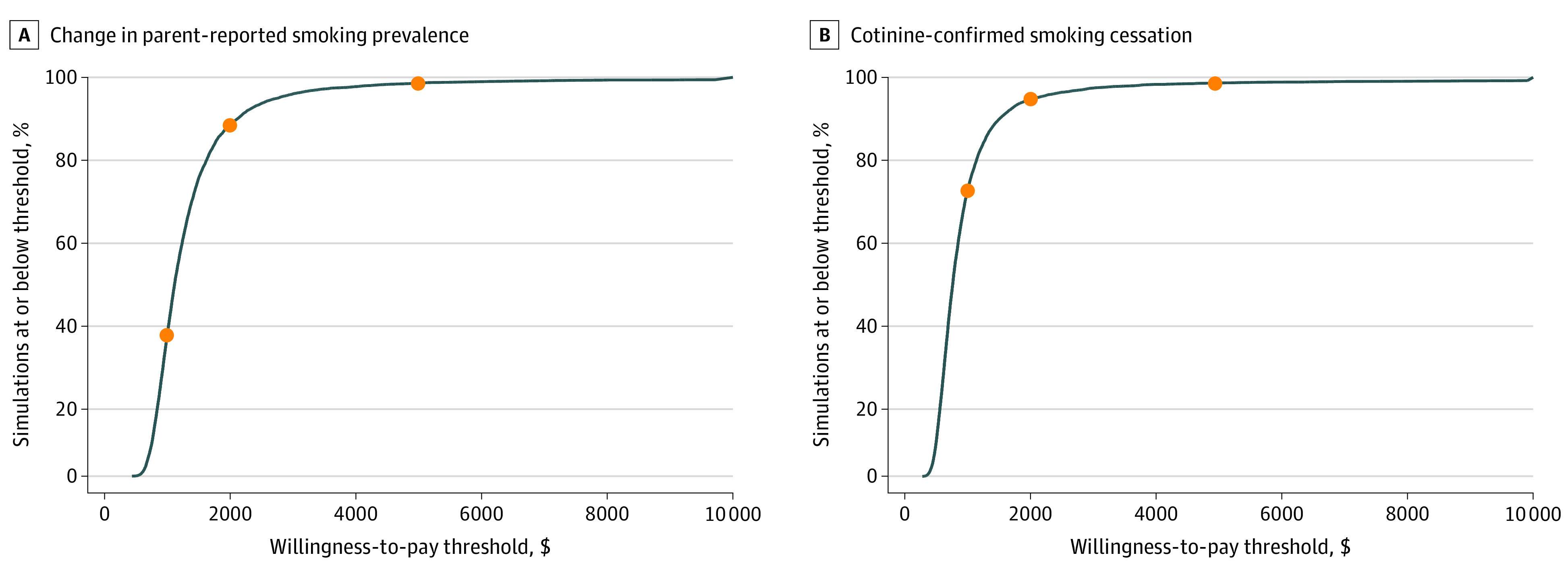
Cost-effectiveness Acceptability Curves Graphs show change in parent-reported smoking prevalence (A) and cotinine-confirmed smoking cessation (B). These graphs represent the percentage of simulations in which the program would be considered cost-effective, according to different willingness-to-pay thresholds taken by a health care organization. For example, in panel A, if a health care organization was willing to pay $2000 per parent-reported quit, simulation results show that there is an 88.0% likelihood that the intervention would have an incremental cost-effectiveness ratio below $2000 per quit. Circles indicate willingness-to-pay thresholds at $1000, $2000, and $5000.

Figure 1 presents cost acceptability curves illustrating the proportion of simulations in which CEASE would be considered cost-effective across a range of different willingness-to-pay thresholds. For example, if a health care organization was willing to pay up to $2000 per quit, the program would be cost-effective in 88.0% of simulations based on effectiveness estimates generated from changes in parent-reported smoking prevalence and 94.6% of simulations based on increased cotinine-confirmed smoking cessation.

Removing costs incurred by a practice that hired a person to follow up with families, as well as costs related to UMass TTS training (which had limited uptake in all but 1 practice), reduces total costs to $97 607 (95% CI, $84 725-$122 549). Using this revised cost, the ICER is $954 (95% CI, $546-$3073) per additional parent who becomes a nonsmoker, a 15% decrease.

**Figure 2.**
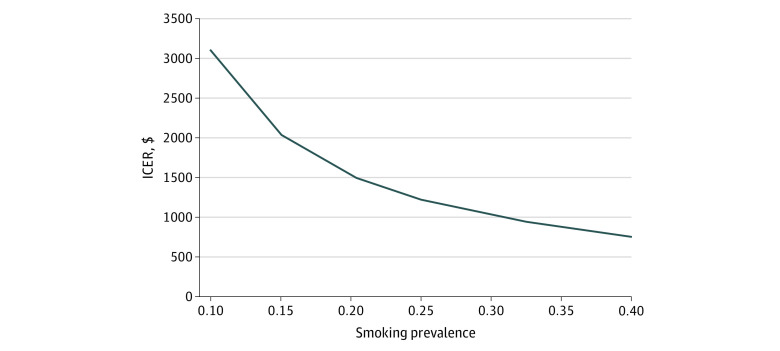
Incremental Cost-effectiveness Ratio (ICER) by Varying Baseline Smoking Prevalence ICER is shown as cost (2018 US dollars) per change in smoking prevalence.

Sensitivity analyses also evaluated the impact of varying smoking prevalence, as well as the duration of the program. If the practice mean smoking rate is 10%, the ICER would be $3068 (95% CI, $1768-$9765) per additional parent who becomes a nonsmoker. Alternatively, if the mean practice smoking rate is 40%, the ICER would decrease to $767 (95% CI, $442-$2441) per additional parent who becomes a nonsmoker (Figure 2).

**Figure 3.**
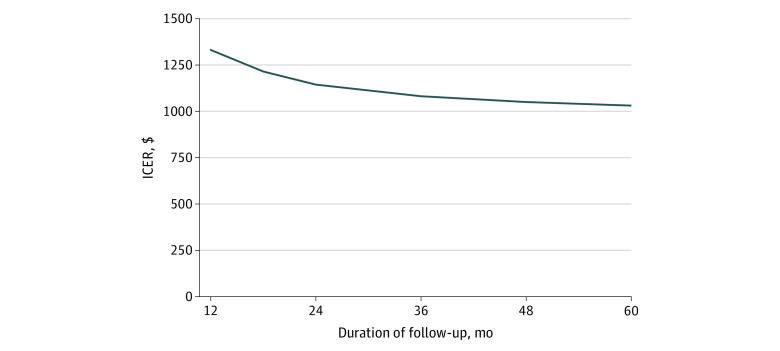
Incremental Cost-effectiveness Ratio (ICER) by Duration of Follow-up, in Months ICER is shown as cost (2018 US dollars) per change in smoking prevalence.

Total costs were evaluated over the 2 years in which the study was conducted. Varying the duration of the program would influence the ICER because the relative contribution of the fixed costs to the total cost would be changed. A program in place for 1 year would have total costs of $66 844 (95% CI, $59 785-$79 720) and an ICER of $1307 (95% CI, $754-$4185) (assuming an effectiveness rate of the same magnitude as that observed over the 24-month study period). Alternatively, a program in place for 4 years would have an ICER of $1032 per quit (95% CI, $593-$3307) (Figure 3), a reduction of approximately 9% compared with a 2-year program.

## Discussion

We estimated the cost per quit of implementing a smoking cessation program for parents whose children were seen in pediatric practices. The analysis used a health care organization’s perspective because, for most practices, it is ultimately those organizations that will decide whether to adopt such a program. Although it is possible for pediatric clinicians who provide parents with cessation services to bill payers for the service, few do.^14^ Some pediatric practices in the CEASE study reported insurer nonpayment or that the amount of effort required to secure payment exceeded the amount of the reimbursement itself. However, to the extent that payment models such as accountable care organizations incentivize improvements in population health, they may prompt systems to reexamine how best to allocate limited resources to maximize health gains.

In the present analyses, the CEASE intervention is estimated to have an ICER of $1132 per parent who became a nonsmoker, compared with usual care. This number compares favorably with other health system–based smoking cessation interventions focused on adults after their published ICERs are adjusted to 2018 dollars using the US gross domestic product deflator.^15,16^ A population-outreach strategy in adults using an electronic health record (EHR)–based smoker registry to connect smokers with cessation resources showed an incremental cost per additional quit of $4137 (95% CI, $2671-$8460), or $4674 (95% CI, $3018-$9559) per quit in 2018 US dollars.^17^ Rigotti et al^18^ evaluated the cost-effectiveness of direct-to-smoker outreach to offer free and easily accessible tobacco treatment (NRT and state quitline) and found an ICER of $464 per quit ($554 per quit in 2018 dollars). Mundt et al^19^ evaluated the cost-effectiveness of a smoking cessation intervention paying Medicaid recipients who smoked to take calls from a tobacco quit line. They calculated the ICER of this intervention as $2480 (95% CI, $1694-$4573) in 2018 dollars per additional smoker who quit.^19^ Curry et al^20^ and Schauffler et al^21^ evaluated the effect of differential insurance coverage for behavioral program and nicotine-replacement therapy and found ICERs ranging from $797 (with standard coverage)^20^ to $1171 (with full coverage)^21^ per quit ($1435 and $2108, respectively, in 2018 dollars).

Sensitivity analyses showed that the CEASE intervention would likely be considered cost-effective for health care systems willing to pay $2000 per quit. Furthermore, these estimates are conservative; they included costs that were not part of the intervention design. Removing those costs from the analysis led to a 15% decrease in ICER from $1132 to $954. It is important to note that wages, estimated using national averages, constituted 88% of the total cost of the intervention in the main analysis. Accordingly, the ICER for any particular health system will be sensitive to how local wages compare with national averages.

As the number of parent smokers identified with effective screening in the practices increases, the incremental cost per quit will decrease, such that practices with nearly double the number of smokers would be expected to have ICERs that are reduced by a third. Specifically, assuming a similar treatment effectiveness but varying smoking prevalence led to large changes in the ICER; as smoking prevalence increased from 10% to 40%, the cost per quit decreased from $3068 to $767. Although the present analysis suggests that CEASE provides an excellent return on investment in almost any pediatric setting in the US, it would be especially cost-effective in low-income communities, which typically have higher smoking rates. Furthermore, although a 40% smoking prevalence is quite high, similar value could also be achieved by more efficiently identifying parent smokers, assuming this required minimal resources. These analyses also illustrate that ICERs are reduced by approximately 9% when initial investments in staff training and infrastructure are amortized over 4 years instead of 2.

Following the publication of the CEASE trial results, concerns were expressed about the financial sustainability of the intervention if it were rolled out more broadly.^9^ This preplanned analysis shows that, although the intervention was conducted as part of a research study, the cost of the programmatic support that could be offered to practices outside the research setting would not undermine the cost-effectiveness of the CEASE intervention if it were deployed broadly. This model of dissemination, with external support, has been adopted by the North Carolina Department of Health and Human Services and the Indiana Chapter of the American Academy of Pediatrics. The CEASE team trained program coordinators in both states, trained the pediatric office staff in how to implement CEASE, and provided ongoing programmatic support, outside the context of a research study.

Certain costs were excluded from the analyses. Increases in clinicians’ interaction time with patients to advise and assist with tobacco cessation were not included because there is no strong reason to believe this would change the number of visits practitioners have with patients and, therefore, adversely affect their revenue. Other economic impacts would likely favor adoption of the intervention. We did not attempt to measure changes in health care expenditures for children whose parents stop smoking. Limited available evidence suggests possible short-term savings, but estimates are small or have weak statistical evidence.^22,23^ In addition, this study did not include changes to parental outcomes aside from cessation itself. For example, parental smoking cessation is associated with improved health and longevity,^1^ reduced health care utilization,^1^ and lower health care expenditures^24^ for the parents themselves.^25^ Parents who quit are also less likely to have a subsequent child born prematurely or small for gestational age,^25^ which may require an extensive hospital stay in a neonatal intensive care unit. Accountable care organizations that cover both children and parents would reap these benefits. When parents quit smoking, children’s exposure to secondhand and thirdhand tobacco smoke is reduced, leading to better health^1,26^ and educational outcomes.^27^ Although they are difficult to quantify, these benefits would increase the favorability of the CEASE intervention compared with usual care.

### Limitations

These cost-effectiveness results are based on the experience of the 10 CEASE trial practices and may not be broadly generalizable. In addition, 1 sensitivity analysis assumed that some practices’ larger personnel investments did not lead to substantial increases in effectiveness. However, the CEASE trial was not powered for such analyses, so further research is necessary before that assumption can be verified.

In the current technological landscape, it is likely that a health system deciding to deploy the CEASE intervention would integrate features to facilitate the delivery of the intervention in its EHR. The study team determined from qualitative interviews with practice leaders that some practices in the intervention group made minor changes to their EHR to facilitate CEASE implementation. A more robust EHR that streamlines CEASE processes might be achieved at the health care organization level and could reduce the overall cost of CEASE greatly, especially if it were then deployed across a large number of practices. Another trial examining the effectiveness and cost-effectiveness of EHR modification to include CEASE is currently under way.^28^

## Conclusions

The CEASE cluster randomized clinical trial was associated with a 3.7% difference in parent-reported smoking prevalence rate among parents attending pediatric primary care compared with usual care practices, at a cost conservatively estimated to be $1132 per parent who became a nonsmoker. This cost-per-quit compares favorably with other smoking cessation interventions in the clinical setting and suggests that the CEASE intervention could be broadly disseminated.

## Notes

Supplement. **eTable 1.** Wage **eTable 2.** Detailed List of Cost Items **eTable 3.** Types of Distribution and Variability Assessment Method for Each Cost Item **eFigure.** Scatter Plots of Incremental Effectiveness (%) and Incremental Cost per Smoker Illustrating How the Cost (Numerator) and Effectiveness (Denominator) Values Vary With One Another 10,000 Probabilistic Sensitivity Analysis Simulations
