# Supplementary material for: Cost-effectiveness of a Smoking Cessation Intervention for Parents in Pediatric Primary Care
Source: JAMA Netw Open. 2021 Apr 1;4(4):e213927. doi: 10.1001/jamanetworkopen.2021.3927 (PMC8017473; doi:10.1001/jamanetworkopen.2021.3927)
Supplement: Supplement. — eTable 1. Wage eTable 2. Detailed List of Cost Items eTable 3. Types of Distribution and Variability Assessment Method for Each Cost Item eFigure. Scatter Plots of Incremental Effectiveness (%) and Incremental Cost per Smoker Illustrating How the Cost (Numerator) and Effectiveness (Denominator) Values Vary With One Another 10,000 Probabilistic Sensitivity Analysis Simulations [file jamanetwopen-e213927-s001.pdf]

Supplemental Online Content

Drouin O, Sato R, Drehmer JE, et al. Cost-effectiveness of a smoking cessation intervention for parents in pediatric primary care. *JAMA Netw Open*. 2021;4(4):e213927. doi:10.1001/jamanetworkopen.2021.3927

eTable 1. Wage
eTable 2. Detailed List of Cost Items
eTable 3. Types of Distribution and Variability Assessment Method for Each Cost Item
eFigure. Scatter Plots of Incremental Effectiveness (%) and Incremental Cost per Smoker Illustrating How the Cost (Numerator) and Effectiveness (Denominator) Values Vary With One Another 10,000 Probabilistic Sensitivity Analysis Simulations

This supplemental material has been provided by the authors to give readers additional information about their work.

© 2021 Drouin O et al. *JAMA Network Open.*

eTable 1: Wage

Occupation title	Corresponding occupation title in Bureau of Labor Statistics	Hourly wage + fringe
Senior Pediatrician	Pediatricians, General; 75th+ percentile	$133.00
Pediatrician	Pediatricians, General	$109.06
Project Manager		$66.50
Research Assistant		$15.96
Registered Nurse	Registered Nurses	$45.86
Nurse Practitioner	Nurse Practitioners	$68.44
Practice Manager	Office manager (AAP)[1]	$33.68
"Practice Staff"	Receptionists and Information Clerks - Office of physicians	$18.63
Medical Assistant	Medical Assistants	$21.49

[1]: Wage for this occupation comes from suggested guidelines from the American Academy of Pediatrics

© 2021 Drouin O et al. *JAMA Network Open.*

eTable 2: Detailed List of Cost Items

Cost	Random component	Fixed component
**Fixed costs**		
Pre-implementation		
Peer-to-peer training	wage, total costs (site-specific)	# workers, # minutes
Training video viewing time	wage, % of video watched	# minutes of video time, # workers
Whole office training call	wage, total costs (site-specific)	# minutes of call time, # workers
Tablet computers		# tablets, # 4G network extender
UMass TTS Training	wage, total costs (site-specific)	Registration cost, # workers, # hours of training time
Post-implementation		
Check-in call (1st month)	wage, # minutes call	# calls, # workers
Pharmacy calls	wage, # minutes call, # pharmacies	
Pharmacy letters	wage, # pharmacies	price of postage, price of envelope/printing
**Variable costs**		
Monthly report preparation	wage, # minutes call	# calls, # workers
Tablet computers data plan		# tablets
Check-in calls	wage, # minutes call	# calls per month, # workers
In office tablet management (with parents)	wage, # minute per smoker, # minutes per non-smoker, % smokers	# days, # workers
In-office tablet management (end of each day)	wage, # minutes per day	# days, # workers
Parent letters	wage, # minutes from RA	price of postage, price of envelope/printing, # letters
Parent calls (one practice only)	wage, # hours call per week	# weeks
Quitline report management time	wage, # minutes	# report per year, # years, # workers
One-year whole office call	wage, total costs (site-specific)	# workers
Programmatic support	wage, # minutes	# months, # workers
Notes: Variation in wages is determined by a wage multipler. The wage multipler was made under the assumption that wages for all job types vary together (for example, due to local economic conditions).		

© 2021 Drouin O et al. *JAMA Network Open.*

eTable 3: Types of Distribution and Variability Assessment Method for Each Cost Item

Category	Item	Type of distribution used	Standard deviation observed vs. assumed
**General**	wage	normal	assumed
**Fixed costs**			
Pre-implementation			
Peer-to-peer training	total costs (site-specific)	normal	observed
Training video viewing time	% of video watched	uniform	assumed
Whole office training call	total costs (site-specific)	normal	observed
UMass TTS Training	total costs (site-specific)	normal	observed
Post-implementation			
Check-in call (1st month)	# minutes call	normal	assumed
Pharmacy calls	# minutes call	normal	assumed
Pharmacy calls / Pharmacy letters	# pharmacies	normal	assumed
**Variable costs**			
Monthly report preparation	# minutes call	normal	assumed
Check-in calls	# minutes call	normal	assumed
In office tablet management (with parents)	# minute per smoker	log normal	observed
	# minutes per non-smoker	log normal	observed
	% smokers	normal	assumed
In-office tablet management (end of each day)	# minutes	normal	assumed
Parent letters	# minutes from RA	normal	assumed
Parent calls (one practice only)	# hours call	normal	assumed
Quitline report management time	# minutes	normal	assumed
One-year whole office call	total costs (site-specific)	normal	observed
Programmatic support	# minutes	normal	observed

© 2021 Drouin O et al. *JAMA Network Open.*

eFigure: Scatter Plots of Incremental Effectiveness (%) and Incremental Cost per Smoker Illustrating How the Cost (Numerator) and Effectiveness (Denominator) Values Vary With One Another 10,000 Probabilistic Sensitivity Analysis Simulations

Panel A: Change in parent-reported smoking prevalence

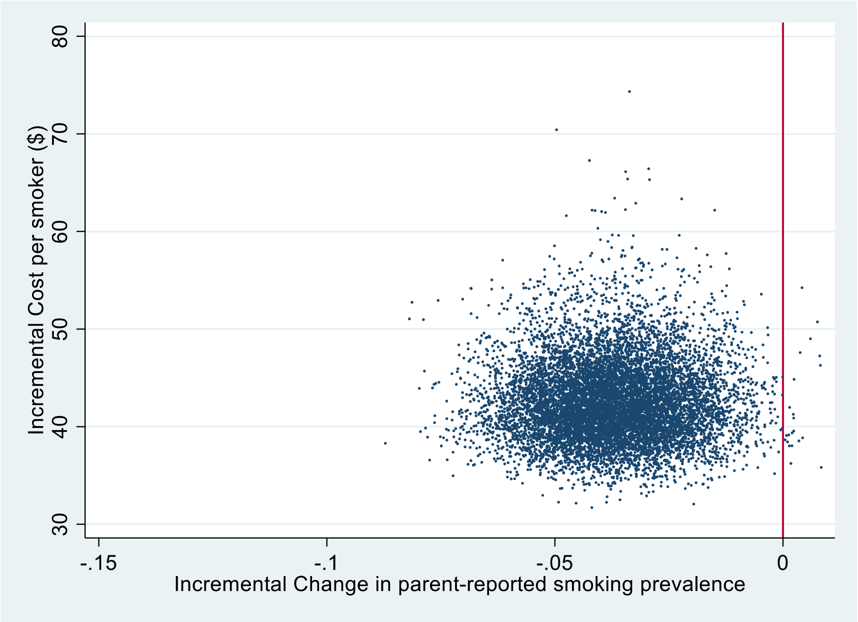

Panel B: Cotinine-confirmed smoking cessation

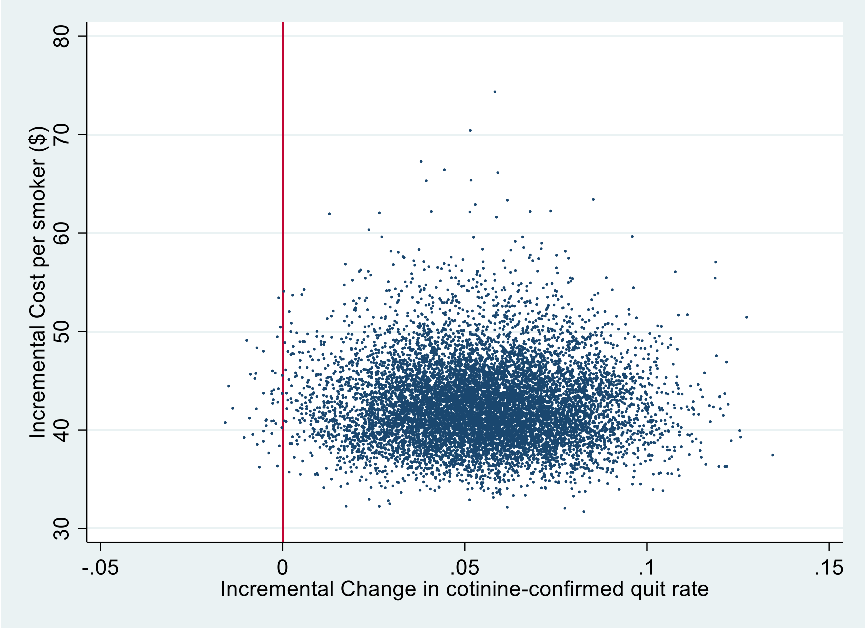

These graphs represent the joint 95% confidence intervals on both incremental effectiveness and incremental cost per smoker.

© 2021 Drouin O et al. *JAMA Network Open.*
